# Telephonic Triage in Times of COVID-19: Experiences at a Telemedicine Center in India

**DOI:** 10.1017/dmp.2021.126

**Published:** 2021-04-23

**Authors:** Avik Ray, Swati Sharma, Balakrishnan Sadasivam

**Affiliations:** Department of Pharmacology, All India Institute of Medical Sciences Bhopal, Bhopal, Madhya Pradesh, India

**Keywords:** COVID-19, public health, telemedicine, telephonic triage, telehealth

Telemedicine and telehealth have played major roles in combating the coronavirus disease (COVID-19) pandemic, opening up newer opportunities for this technology-laden wing of medicine.^1,2^ We hereby put forward the experiences from a telemedicine center in India, while forecasting whether it could be used as a preparedness tool for future pandemics, based on our observed benefits.

At the All India Institute of Medical Sciences (AIIMS) Bhopal, India, the telemedicine center had been assigned the task to receive calls over a toll-free number 24/7, starting March 23 through December 10, 2020.^3^ The resident doctors from the Department of Pharmacology had been deployed on a rotational basis to do triage by telephone regarding COVID-19 testing. We followed the regularly updated guidelines of the Indian Council of Medical Research (ICMR) to categorize and prioritize patients.^4^ Initially, the triage was mainly based on their travel history – although, after the nationwide lockdown was imposed, we adopted a symptom-based approach where we asked in detail whether there were symptoms of acute respiratory illness or possible contact with a COVID-19 patient.

Our main objective was to assess whether the patients needed to come for testing on a case-by-case basis and hence, decrease the unnecessary hospital footfalls. This was primarily in view of the scarcity of testing kits in India in the initial few months of the breakout of the pandemic.

**Figure 1.**
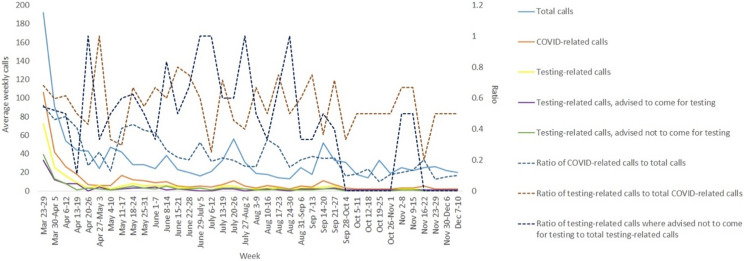
Trends of calls received at the telemedicine center between March 23, 2020, and December 10, 2020. A few critical and relevant ratios are also illustrated.

We received, in total, 1302 calls that included both COVID-19 and non-COVID-19-related inquiries; 80% of the callers belonged to the age range of 25–35 years. Figure 1 shows the trends of calls received. It is evident that the number of calls declined gradually over the course of time, starting with 192 in the first week and dipping to as low as 13 in the last week of August. The total number of COVID-19-related calls was 381, of which 59% was testing-related. The ratios illustrated in the figure indicate that the proportion of calls related to COVID-19, as well as testing-related calls, declined gradually, which could be elucidated by the withdrawal of lockdown and resumption of the institute OPD (Out-Patient Department) services. The ratio of testing-related calls where we had advised not to come for testing to the total number of testing-related calls serves as a marker for our objective, since it indicates whether we were able to reduce avoidable hospital visits. As the figure depicts, this proportion was quite high till the end of August, followed by a gradual decline, which could be due to relaxation of testing criteria, availability of sufficient number of RT-PCR testing kits, emergence of rapid-antigen testing kits, and a greater degree of awareness among the people regarding the need for testing. Those who had called for inquiring about testing but were asked not to come, were given proper consultation for their symptoms and were advised appropriate over-the-counter medications. In case of any confusion, residents consulted the senior physicians. No special referral codes were given to callers advised to come for testing. Among the non-COVID-19-related calls, the major categories (proportions) were OPD inquiries (10%), interstate travel (5%), quarantine information (10%), queries related to chronic conditions and ongoing medications (20%), and others (5%).

With the help of this telephonic triage, it was possible to attain our objective of curtailing the unnecessary footfalls in our hospital, and hence reducing the non-essential face-to-face interactions. More importantly, it ensured optimal use of testing kits.

In conclusion, to contain a pandemic, telemedicine preparedness should be stressed upon and incorporated as a mainstream health care service delivery platform, allowing us to use it as the first mode of consultation for even commonly encountered infectious diseases, such as seasonal flu. Although the human touch is lost, this would be a humane decision in such scenarios.
